# Cutaneous Fungal Infections in Patients Experiencing Homelessness and Treatment in Low-Resource Settings: A Scoping Review

**DOI:** 10.7759/cureus.30840

**Published:** 2022-10-29

**Authors:** Taha F Rasul, A. C Gamret, Orly Morgan, Daniel R Bergholz, Emily Eachus, Megan Mathew, Arfa Faiz, Adam Elkhadem, Victoria Dahl, Gabriel Motoa, Sana Gulraiz, Armen Henderson, Brian W Morrison

**Affiliations:** 1 Department of Dermatology and Cutaneous Surgery, University of Miami Miller School of Medicine, Miami, USA; 2 Department of Public Health, University of Miami Miller School of Medicine, Miami, USA; 3 Department of Allergy and Immunology, Sutter Medical Center, Sacramento, USA; 4 Department of Public Health, Columbia University, New York City, USA; 5 Department of Internal Medicine, University of Miami Miller School of Medicine, Jackson Memorial Hospital, Miami, USA; 6 School of Public Health, West Virginia University School of Medicine, Morgantown, USA; 7 Department of Internal Medicine, University of Miami Hospital, Miami, USA

**Keywords:** patients experiencing homelessness, low resource, cutaneous fungal infections, scoping study, homelessness

## Abstract

Patients experiencing homelessness (PEH) suffer from a high burden of cutaneous fungal infections. Preventative treatment is important as such infections can lead to harmful complications such as cellulitis and even osteomyelitis. There are sparse data regarding cutaneous fungal infections of homeless populations and management in low-resource settings. A MEDLINE search was conducted using the key terms “cutaneous,” “fungal,” “infections,” “dermatophytes,” and “homeless.” The search included case-control, cohort, and randomized controlled trials published in the English language. This scoping review of studies yielded information with regard to practical treatment advice for providers in low-resource settings, including medical, hygiene, prevention, and treatment options for PEH with cutaneous fungal infections, the most common of which were tinea pedis (3-38%) and onychomycosis (1.6-15.5%). Few studies have been conducted on the differences between sheltered and unsheltered homeless patients, which can have treatment implications. Systemic antifungal therapy should be carefully considered for diffuse, refractory, or nail-based cutaneous fungal infections if there is a history of alcohol use disorder or liver disease. While PEH have a high risk of alcohol use disorder, this can make definitive treatment challenging.

## Introduction and background

Cutaneous fungal infections (CFIs) affect roughly 25% of the world population [1]. In the United States, CFIs comprised approximately 4 million visits to medical providers from 1995 to 2004 [2]. Although many CFIs can follow an indolent course in immunocompetent individuals, they can also predispose patients to epidermal dysfunction and become a nidus for superimposed infections. Patients experiencing homelessness (PEH) form a group particularly vulnerable to CFIs and CFI-related complications such as cellulitis and osteomyelitis [1-3]. This can be attributed to a multitude of causes, including increased rates of traumatic injury, chronic illnesses, malnutrition, and decreased access to reliable healthcare services.

PEH often face barriers to adequate care, particularly in the traditional clinic setting. To address some of these barriers, providers have established mobile clinics, which allow for the evaluation of CFIs in a street setting. Limited funding and volunteer-based staffing mean that such clinics operate in low-resource conditions [4]. CFI management for low-resource providers is therefore an important consideration for overall skin health and requires further elaboration [4]. Additionally, studies conducted on homeless populations thus far have primarily focused on sheltered cohorts, leaving open questions about the health of unsheltered PEH [1].

This paper reviews common dermatophyte infections found in PEH by anatomic location, covers main clinical presentations, discusses further avenues for research, and offers literature-based recommendations on managing such conditions for street medicine and resource-limited providers.

## Review

Methods

Overview

A MEDLINE literature review was conducted using the terms “dermatophyte,” “unsheltered,” “fungal,” “infection,” “tinea,” and “homeless.” Studies included randomized controlled trials, meta-analyses, and observational studies. The review was prepared using the PRISMA-ScR checklist. The search was restricted to English-language articles. Photographs of conditions were included from an author’s (B.M., MD) clinical encounters (both in a traditional clinic and volunteer street medicine clinic) as well as peer-reviewed open-access publications. In order to demonstrate the average clinical presentation of each disorder, deidentified photographs collected of patients encountered in the street and clinical setting were obtained with their explicit permission. Written consents and surveys were filled by patients and reviewed by research staff. Patients were fully informed that their conditions would be used for educational and research purposes.

Search Strategy and Information Sources

A literature search was performed in MEDLINE including articles published between January 1, 1979, to January 1, 2022, and included the following search terms:

Homeless Persons OR Homeless *OR Unsheltered OR Street people *OR Housing instability OR Housing instability OR Vulnerable populations) AND (Derm OR Skin* OR Dermatophytosis OR Fungal skin infection OR Tinea OR Cutaneous fung OR Dermatophyt OR Fungal infection

Inclusion Criteria

Original research, case series, and case reports mentioning CFIs in housed or unhoused populations were included. Inclusion criteria were (i) studies regarding sheltered or unsheltered PEH, (ii) studies containing epidemiologic data on tinea pedis or cutaneous fungal disease directly or in association with other skin or systemic conditions, and (iii) CFI management strategies in resource-limited settings. Preliminary reports, commentaries, and conference abstracts were also added if found by the study team to have appropriate information for the review.

Exclusion Criteria

Articles including infectious skin disease without mention of fungal or superimposed infections were excluded. Articles that did not mention homelessness in regard to fungal infections were also excluded if they did not otherwise evaluate mechanisms of fungal transmission. By this method, articles would fall into two broad categories: articles specifically dealing with cutaneous fungal disease in PEH, and articles providing background on fungal pathophysiology in general.

Article Selection, Data Extraction, and Analysis

**Figure 1:**
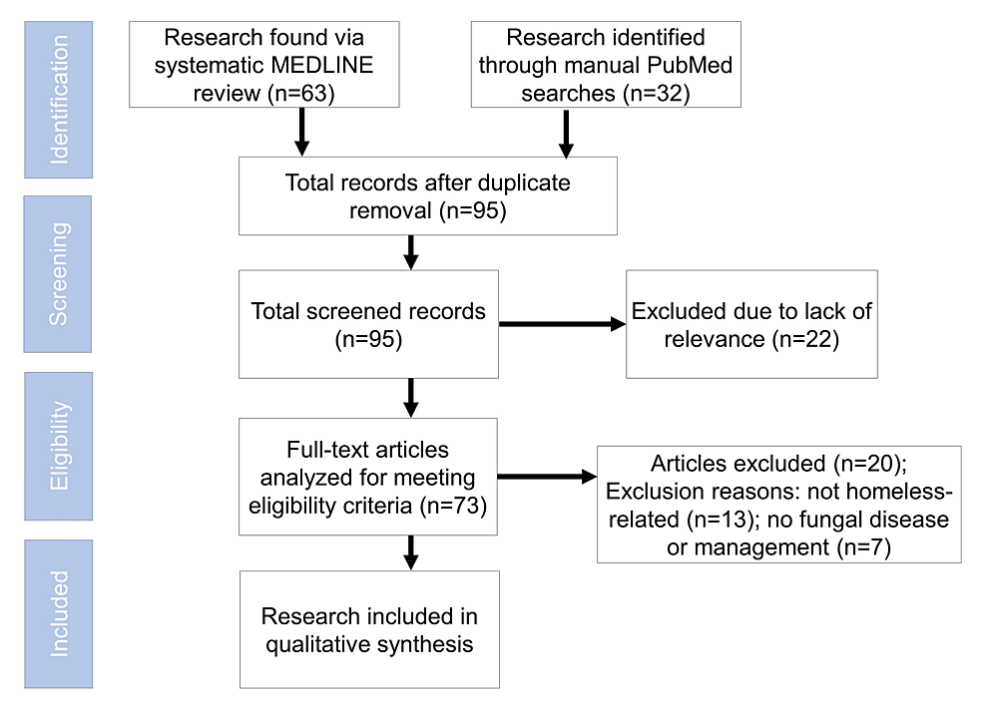
Flowchart of article selection, removal, and inclusion into the final review.

In total, 53 articles met the inclusion criteria (Figure 1). Two reviewers (T.R., D.B.) completed the preliminary review of abstracts to see whether articles met the inclusion criteria. In cases where it was unclear, a third reviewer (A.C.G.) was used for arbitration. Next, all included studies were read, and data pertaining to CFI parameters were collected, as well as demographic characteristics, diagnoses, outcomes, and significance of study. Peer-reviewed articles evaluated for photographs of conditions were not analyzed using this process.

Assessing Study Quality

T.R. and D.B. determined study quality via the Oxford Center for Evidence-Based Medicine Levels of Evidence Scale [5]. Study quality was rated on a numerical scale from 1 to 5, with 1 being the lowest quality and 5 being the highest. Level of evidence was also rated from A (highest level) to D (lowest level). The article was then prepared using the PRISMA extension for scoping review guidelines [6].

Results

Methodological Quality of Works Assessed

**Figure 2:**
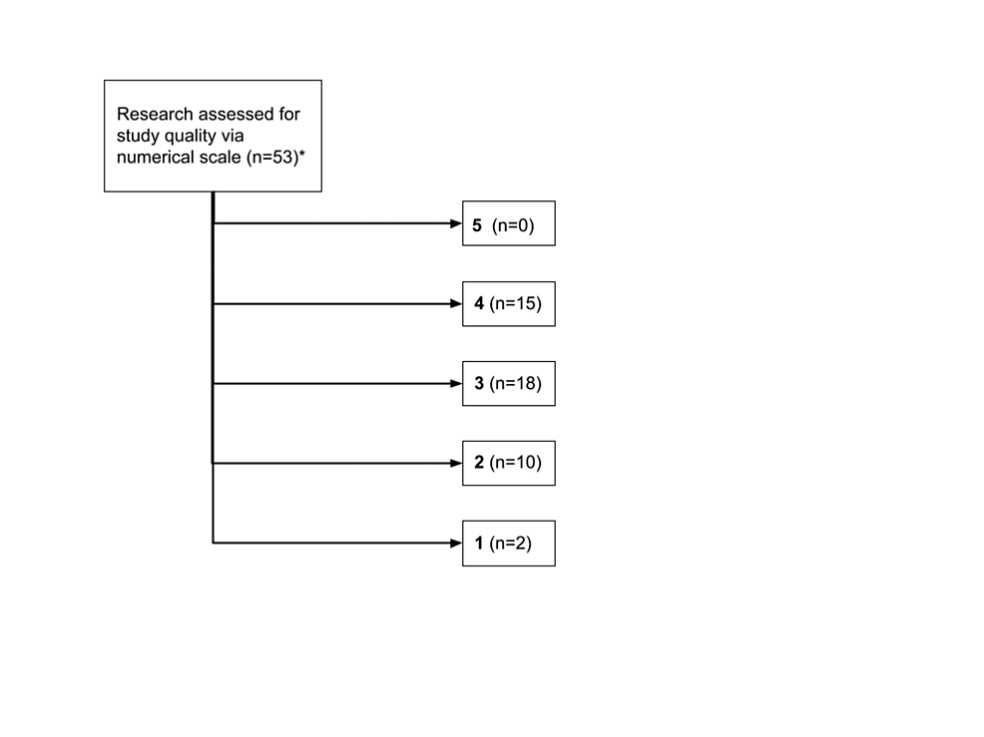
Numerical scale applied to study quality of works assessed. A total of eight articles were determined to sufficiently meet the eligibility criteria, even though they were not assigned a numerical scale. These included checklists, reports with pictures, and basic sciences papers.

In the primary literature, methods were considered moderate with a mean and median of 3 (Figure 2). The studies demonstrated heterogeneity in regard to homeless populations (shelter-based or otherwise), inclusion criteria, follow-up period, and method of patient examination [5,6]. A few studies demonstrated insufficient validity to be included in a scoping review of PEH.

Among articles reviewed, the prevalence ranges of CFIs were noted (Table 1).

**Table 1:** Prevalence ranges of cutaneous fungal infections among PEH PEH, patients experiencing homelessnes

Anatomic Region	Disease	Frequency
Head, hair, and face	Tinea capitis	0.4-5% [7,8]
	Tinea barbae	Few reported cases [9-14]
Torso and proximal extremities	Tinea corporis	0.4-7.1% [10,11]
	Tinea cruris	0.7-1.6% [11,12]
	Pityriasis versicolor	0.4-2.1% [10-13]
Distal extremities	Tinea pedis	3-38% [7-12]
	Tinea manuum	0.7-0.9% [13,14]
	Onychomycosis	1.3-15.5% [7-14]

Tinea Capitis

Tinea capitis is a contagious fungal infection of the scalp, hair follicle, and hair shaft. It is generally spread person-to-person but can also be acquired by fomite transmission as well as zoonotically [7-9]. The overall prevalence of tinea capitis has increased over the past two decades [9,10]. Afro-textured hair has a higher risk of tinea capitis infection, and, as such, there is a greater prevalence in black communities [11,12].

Tinea capitis can have a diverse set of presentations and often begins with scaly papules that increase in size over time [13,14]. Tinea capitis infections can manifest as circumscribed areas of hair loss with visible follicles due to the fungal invasion, weakening the hair keratin and causing breakage. A late-stage complication of tinea capitis is permanent hair loss (scarring alopecia), which typically occurs in kerion, the inflammatory clinical type [15-17]. Kerion is also associated with purulent discharge, which can cause scarring alopecia. A sensitive sign on physical examination is cervical lymphadenopathy, which can be appreciated in both inflammatory and non-inflammatory types [18-22].

Commonly used drugs include griseofulvin with a four- to eight-week course and terbinafine with a six-week course [23,24]. Although both drugs have similar efficacy and safety profiles, griseofulvin is often preferred in low-resource settings because it is the most cost-effective form of treatment [14].

A retrospective study by Shahriari et al. found less than 5% of patients evaluated had tinea capitis [11]. A study based in Belgium found tinea capitis in one patient out of a cohort of 337 but did not specifically elaborate on the overall prevalence in homeless populations [25]. While these studies would show tinea capitis to not be a large problem in the PEH population, the low incidence rates may stem from lack of data rather than lack of cases. There may also be geographic variation, and studies conducted in cities with larger African American populations would likely show more tinea capitis.

Tinea Barbae

**Figure 3:**
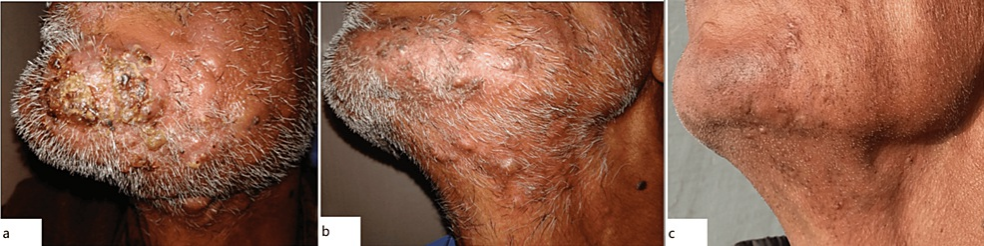
Tinea barbae with kerion on the chin. Notice inflammatory plaques with pustules and crusts. Initial presentation (a) was followed by evaluation at three weeks of oral terbinafine (b) and eventual resolution with scarring at six months (c). This figure has been taken from an open-access journal under a CC-BY license. Source: Indian Journal of Dermatology, Venereology, and Leprology [27].

Tinea barbae is a rare dermatophytosis that involves the hair and skin around the beard (Figure 3). Spread occurs from animal to person, and less commonly from person to person. Autoinfection in patients with pre-existing tinea infections on other areas of the body (for example, tinea pedis) has also been documented [26,27]. The presence of health factors such as immunosuppression, diabetes mellitus, and a history of dermatophyte infections can increase the risk of developing tinea barbae infections.

Reliable methods of diagnosis include direct microscopic examinations of potassium hydroxide (KOH) skin scrapings, with fungal cultures and skin biopsies used for refractory cases. The incision and drainage of tinea barbae lesions have shown limited therapeutic benefit [17,28-31]. First-line treatment is oral antifungal therapy, with topical medications used as adjuncts, including terbinafine and ketoconazole. The presence of tinea barbae in homeless populations has been limited to a few reports, primarily reported by providers in homeless shelters [19]. PEH may be at higher risk for tinea barbae infections due to close living quarters in shelters, which may further facilitate the spread of this disease. For PEH populations, tinea barbae poses particular concern for the potential complication of secondary bacterial infection. PEH are at an increased risk for MRSA colonization, particularly around the nares [29]. If a patient does present with tinea barbae, they should receive treatment immediately and be connected to subsidized medical services for prevention of spread [26].

Tinea Corporis

In the general population, tinea corporis also has a skin-to-skin method of transmission [18,30]. Close contact with dogs or cats harboring Microsporum canis can also lead to direct disease transmission [31]. Much like other dermatophytoses, tinea corporis tends to present with annular, scaly, red plaques with central clearing [32,33]. The diagnosis of tinea corporis is clinical, but KOH preparation can be done for confirmation [34-37]. Treatment is primarily in the form of topical medications such as azoles (clotrimazole, miconazole) as well as terbinafine. Oral therapy is generally reserved for widespread infections and includes oral terbinafine, itraconazole, or griseofulvin.

Zakaria et al. found that 7.1% of all PEH encountered had at least one fungal or non-tuberculous mycobacterial skin infection, including tinea corporis [9]. All other research articles found for this study did not mention epidemiological rates or clinical findings of tinea corporis in PEH populations.

Tinea Cruris

Tinea cruris is a dermatophyte infection that involves the groin area and is also known as “jock itch.” Autoinfection is common, especially in tinea cruris as foot-to-groin spread can occur. Predisposing risk factors include occlusive clothing, improper hygiene, immunocompromised status, and lower socioeconomic status. Excessive perspiration has the strongest association with development of tinea cruris infection, particularly in intertriginous areas [21,38,39]. Clinical symptoms include pruritic rash in the groin area, often with maceration and a light, wrinkled appearance due to excess moisture. This differs from cutaneous candidiasis, which typically has isolated “satellite” lesions in addition to the main, larger rash. Physical examination typically shows red scaly plaques, sometimes with an annular appearance. Infection can be noted anywhere from the perineal region to the upper thigh. Management of tinea cruris includes topical therapies such as terbinafine and azoles. Oral preparations are used to manage refractory or extensive disease [40-46].

The risk and epidemiologic factors of tinea cruris suggest that there would be a high prevalence in humid, tropical environments such as in the Southeastern United States; however, few research studies have been conducted in this geographical area. Studies conducted in homeless shelters in Massachusetts and California recorded the frequency to be between 0.7% and 1.6% [7,11].

Tinea Versicolor

**Figure 4:**
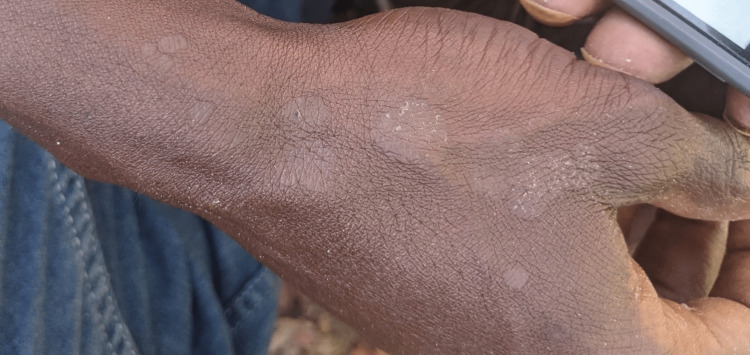
Tinea versicolor of the hand. Pale, segmented patches with scale are present. Deidentified photograph taken directly from author's clinical practice with necessary consent obtained.

Tinea versicolor (also known as pityriasis versicolor) is a CFI caused by a non-dermatophyte Malassezia species. Lesions usually occur on the chest, neck, back, and upper arms. As a normal component of skin flora, Malassezia has increased proliferation in warm and humid conditions [47-50]. For example, the prevalence of infection in colder climates is around 1.1%, whereas it can be as high as 50% in tropical regions [22]. Darker skin types more commonly undergo hypopigmentation due to Malassezia infection, whereas lighter skin types tend to have faint erythema or hyperpigmentation (Figure 4).

First-line treatments include topical preparations with selenium sulfide as well as ketoconazole, both in cream and shampoo form. Ciclopirox has a higher efficacy than ketoconazole, though its high cost makes it resource prohibitive. Oral treatment such as short-term fluconazole and itraconazole can be used for diffuse or refractory lesions [24].

Studies found in the literature review did find results on tinea versicolor. Stratigos et al. [7] and Contag et al. [10] found that tinea versicolor has been observed in cohorts of homeless patients at a frequency ranging from 1.6% to 2.1%.

Tinea Manuum

**Figure 5:**
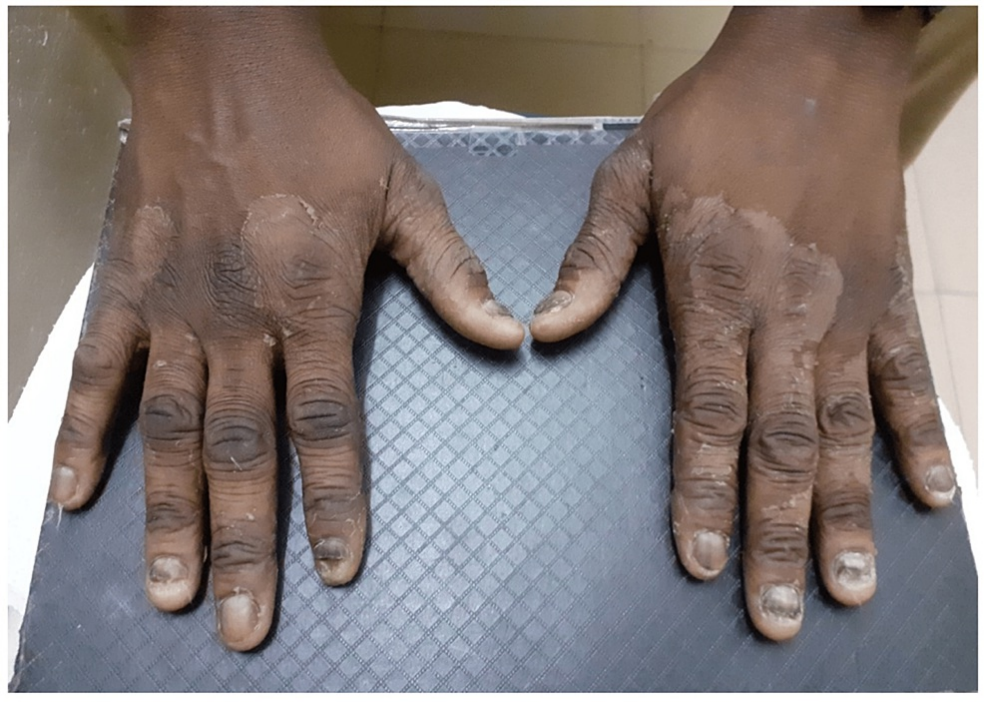
Peeling, dryness, and scale in tinea manuum. There is dorsal and digital involvement as well as increased skin markings. This patient also has concurrent fingernail infection (tinea unguis). This figure has been taken from an open-access journal under a CC-BY license. Source: Journal of Fungi [51].

Tinea manuum commonly involves the palmar aspects or interdigital folds of the hands and can present as thickened skin or pruritis (Figure 5). Tinea manuum can also be transferred via self-infection and by skin-to-skin contact. The dorsum of the hand can present similarly to tinea corporis, with an annular, centrally cleared red plaque. In fact, in some cases, lesions on the dorsum of the hand are classified as tinea corporis rather than tinea manuum. Risk factors are male gender, humidity, and hyperhidrosis [2]. Tinea manuum has been referred to as “two-foot, one hand syndrome” where both feet are affected by tinea pedis and then one hand develops scaly patches or diffuse dryness [28]. There have been very few cases of tinea manuum recorded among PEH within shelters. One study assessed the prevalence of tinea manuum in a shelter in Boston to be approximately 0.7% [7].

Tinea Pedis

The most commonly identified fungal infections identified in the literature include tinea pedis and onychomycosis [28]. It is caused by dermatophyte infection of the feet, including the soles, interdigital clefts, and nails. It has been estimated that approximately 10% of the total population in the United States may suffer from tinea pedis infections [26]. Tinea pedis is a multifactorial disease that is influenced by lifestyle, genetic predisposition, and immunologic susceptibility [14]. Risk factors for infection include humid environments, occlusive footwear, and excessive sweating. Improvement of hygiene conditions can act as prevention of tinea pedis. For example, regular cleaning of swimming pools and bathing areas and frequent washing of change rooms can help in controlling the spread of tinea pedis between individuals [2].

Initial symptoms of tinea pedis include scaling, fissuring, peeling, erosion, and erythema, as well as pruritus and malodor [27-31]. Chronic intertriginous tinea pedis is the most common form of tinea pedis and is commonly seen on the interdigital and plantar digital surfaces of the feet. A thorough history and physical examination are often sufficient to diagnose tinea pedis.

Tinea pedis and onychomycosis greatly increase the risk of developing cellulitis and therefore must be treated promptly [35]. Topical azoles such as miconazole and clotrimazole have a favorable side effect profile and are used widely for treatment [32]. Topical terbinafine has also been shown to produce favorable results in treating tinea pedis. Systemic treatment via oral medication is used when there is involvement of the dorsum of the foot, heel, sole, or refractory infection with blisters [32]. Patients with liver disease such as those suffering from alcohol use disorder are at risk for further hepatotoxicity with systemic terbinafine. Liver enzymes should be measured prior to initiation of oral antifungal therapy in high-risk populations. Medication history is also essential, as systemic antifungals can interact with various medications due to cytochrome p450 effects. For example, oral antifungal therapy cannot be mixed with the antipsychotic pimozide. Together they can have compounding cardiovascular effects, including QT prolongation [36-38]. This is because the typical antifungal course for tinea pedis is only two weeks, whereas most liver transaminase abnormalities occur at six weeks of treatment [32].

More research has been done on tinea pedis in PEH populations compared to other fungal infections. A study by Zakaria et al. found that among PEH at a shelter-based health clinic in the San Francisco Bay Area, approximately 14% of all dermatologic diagnoses included tinea pedis, suggesting a higher prevalence compared to other fungal infections such as onychomycosis and cutaneous candidiasis [9]. Stratigos et al. estimated the prevalence of tinea pedis to be 38% in a Boston-based shelter population [7]. Another study based in two homeless shelters in San Francisco Bay Area found that tinea pedis and foot pain were present in 24% and 56% of participants, respectively [10].

PEH often use shared shower facilities, which increases the likelihood of infection [31]. Additionally, PEH who receive treatment require decolonization of dermatophytes in their footwear to prevent reinfection, which is when there is a lack of access to clean footwear.

Onychomycosis

Infection of the nail with any fungal organism (yeasts, aspergillus, etc.) is known as onychomycosis. The infection can occur in both fingernails and toenails, but onychomycosis of the toenail has a higher prevalence. Approximately half of dystrophic toenails are due to mycotic disease.

Tinea pedis and onychomycosis are closely associated. In most cases, dry and asymptomatic tinea pedis can directly precede the development of onychomycosis. Over time, the closed, moist environment of shoes combined with repetitive microtrauma on the nail while walking can seed the distal nail bed with dermatophytes [31]. Tinea pedis can spread to the skin adjacent to the nail, causing onychomycosis [46]. By extension, tinea pedis can lead to epidermal dysfunction such as skin fissures and predispose a patient to infections by creating an opportunity for further bacterial infections [47,48,51-53].

KOH is a common test that can visualize dermatophytes separate from saprophytic fungi. Tinea cruris, manuum, or psoriasis can also accompany onychomycosis, and therefore areas such as the feet, scalp, and hands should also be examined [42]. Other nail disorders such as trauma injury and nail psoriasis should be included in the differential diagnosis [54-56]. Due to the high risk of cellulitis from onychomycosis (and tinea pedis), treatment is preferable and presents a compelling reason to begin systemic treatment.

If systemic therapy will be used as treatment, confirmatory testing of onychomycosis is recommended in the form of KOH preparations or nail clippings sent to pathology to check for fungal elements [33]. Due to potential hepatotoxicity, a history of hepatitis and alcohol use should be carefully considered before initiation of therapy. There are similar risks to systemic therapy in tinea pedis, albeit with longer treatment times.

While systemic antifungals have side effects, especially in patients with multiple comorbidities or on multiple medications, they are the most effective treatments for onychomycosis. Dermatologists use two different measurements to determine whether onychomycosis has been cured. The “complete cure rate” is a more stringent definition than “mycological cure rate” as complete cure requires negative mycology and 100% clear nails. For example, the complete cure rate of systemic terbinafine is approximately 38%, which is half the mycological cure rate. The mycological cure rate is 48% for systemic fluconazole, 63% for itraconazole, and 76% for terbinafine [34]. Topical therapy is much less effective, with complete cure rates ranging from 8.5% to 18% [34]. Despite thorough treatments, there is generally a high recurrence rate observed, at approximately 5-50%, again likely due to colonized socks and footwear [35,36].

Onychomycosis is one of the more common fungal infections observed in homeless populations, ranging from 1.6% to 15.5% in shelter-based clinical studies. A study by Jones found that among homeless patients, unspecified foot nail pathologies ranged between 15% and 60%, including ingrown toenails and nail fungus [31]. Onychomycosis can be present in multiple toenails with normal nails interspersed.

Discussion

For dermatophyte infections, there are distinct risk factors for infection in both the sheltered and unsheltered PEH populations. Shelters may be crowded and facilitate the spread of skin-to-skin dermatophytoses [38]. Prevention of dermatophyte infection in both sheltered and unsheltered communities is similar and can include hygiene products, clean socks, and new, non-colonized footwear.

Most patients who are not immunocompromised, nor have major underlying chronic disease, can manage their cutaneous mycoses with topical medications such as azoles, terbinafine, griseofulvin, or ciclopirox. Non-dermatophyte mycoses such as Malassezia are also managed with topical ketoconazole, selenium sulfide, or pyrithione zinc [40-43]. In the case of onychomycosis, which also has a high prevalence in the homeless populations studied, the most definitive treatment is systemic. The topical medications can be acquired relatively easily by providers and therefore should be included in CFI management for PEH encountered in street-medicine settings.

Providers need to weigh the costs and benefits of treating tinea infections, particularly in high-risk PEH patients. It is important that providers take a thorough history before providing medications, as many of these medications can have hepatotoxic effects. Alcohol use is frequent in high-risk populations like PEH, and as such there is a high frequency of liver disease among such patients [41].

Therefore, the risk versus benefits of treating PEH with systemic antifungal medicines must be carefully assessed, especially in settings where close follow-up is not possible. The provision of powder-based topical antifungals and clean socks can stop the progression of onychomycosis by reducing the moisture levels and microtrauma to the nails. This can also help treat primary or secondary tinea pedis.

This study has limitations. The literature review was only taken from one online database and only English articles were included. In addition, there is an inherent limitation to reviewing existing literature on PEH populations, as they are an understudied group and as such there is a shortage of available data from which to draw. Additionally, the limited number of articles retrieved in the date range evaluated demonstrates a notable gap in the literature. A larger systematic review or meta-analysis is needed to appraise the true extent of integumentary fungal diseases in homeless populations.

These studies were conducted in either homeless shelters or established medical facilities, whereas there were no studies addressing unsheltered PEH. There are statistically significant differences in the health conditions, healthcare utilization, and overall outcomes between unsheltered and sheltered homeless individuals [37]. As such, there would likely be differences in skin disease burden in the two groups, especially in terms of CFIs.

Homeless Patients and Targeted Skin Examinations: An Area of Further Investigation

The most common fungal skin infections encountered among PEH are tinea pedis and onychomycosis [7-11,19]. Therefore, initial evaluation of PEH could include a brief examination of the interdigital spaces, plantar surface, and toenails. However, there are limited data on associations between fungal infections and other cutaneous diseases in homeless populations. Furthermore, preventative measures in the context of low-resource settings and unsheltered PEH need further elucidation.

Challenges in Diagnosis and Treatment of Onychomycosis

Only half of dystrophic nails are onychomycotic, requiring caution in diagnosis [41,42]. Evaluation by telemedicine dermatologists in conjunction with analysis of nail clippings or KOH preparations could be quick, low-cost measures to guide treatment in low-resource settings. Even so, there are limited data on the efficacy of these diagnostic measures for low-resource providers.

Weighing the Benefits and Risks of Systemic Versus Topical Therapy and Steps for Conservative Management

Most cases of tinea pedis, corporis, cruris, and manuum (except for pustulopapular and diffuse) can be treated topically. There are a few dermatophyte situations such as tinea capitis, tinea barbae, and most cases of onychomycosis, which require systemic therapy [53]. The benefits of treating this condition must carefully be weighed, especially in the absence of appropriate metabolic and liver function panels [22,23,37]. PEH are at a high risk of alcohol use disorder and hepatitis, but CFIs can predispose to recurrent infections, chronic non-healing wounds, and ulcers. It remains to be seen whether there is a net benefit of systemic antifungal treatment compared to the inherent risks of such interventions. Therefore, this also presents a notable area for further research, especially in the field of underserved medicine.

## Conclusions

Findings of this study show a lack of epidemiologic data on skin infections in unsheltered PEH patients, but it did identify that certain CFIs are common in these settings - tinea pedis and onychomycosis - and must be kept in mind when treating PEH. Prevention of CFIs in resource-limited street settings can come in the form of clean socks and hygiene products. Treatment can generally be in the form of topical antifungal medications, with systemic therapy reserved for refractory disease and onychomycosis.

Risk-benefit analysis must be considered when treating patients in low-resource settings, as systemic therapy can have hepatotoxic side effects and require routine monitoring. Previous literature has not focused specifically on fungal skin infections, which can be a major source of morbidity in this vulnerable patient cohort. Such information can expand research directions for resource-limited medical settings.

## Appendices

Supplemental data, filled PRISMA-Scoping Review Checklist, and graphical abstract available at the following repository:

https://osf.io/wfbez/?view_only=2f34986ffd524b3e99a3fec210e6fe1f

## References

[1] Badiaga S, Menard A, Tissot Dupont H (2005). Prevalence of skin infections in sheltered homeless. Eur J Dermatol.

[2] Panackal AA, Halpern EF, Watson AJ (2009). Cutaneous fungal infections in the United States: Analysis of the National Ambulatory Medical Care Survey (NAMCS) and National Hospital Ambulatory Medical Care Survey (NHAMCS), 1995-2004. Int J Dermatol.

[3] Sahoo AK, Mahajan R (2016). Management of tinea corporis, tinea cruris, and tinea pedis: a comprehensive review. Indian Dermatol Online J.

[4] Doohan NC, Mishori R (2020). Street medicine: creating a "classroom without walls" for teaching population health. Med Sci Educ.

[5] (2022). Oxford Centre for Evidence-based Medicine—Levels of Evidence. http://www​.cebm.net/index.aspx?o=1025.

[6] Tricco AC, Lillie E, Zarin W (2018). PRISMA Extension for Scoping Reviews (PRISMA-ScR): checklist and explanation. Ann Intern Med.

[7] Stratigos A, Stern R, González E (1999). Prevalence of skin disease in a cohort of shelter-based homeless men. J Am Acad Dermatol.

[8] Grossberg AL, Carranza D, Lamp K, Chiu MW, Lee C, Craft N (2012). Dermatologic care in the homeless and underserved populations: observations from the Venice Family Clinic. Cutis.

[9] Zakaria A, Amerson EH, Kim-Lim P, Fox L, Chang AY (2022). Characterization of dermatological diagnoses among hospitalized patients experiencing homelessness. Clin Exp Dermatol.

[10] Contag C, Lowenstein SE, Jain S (2017). Survey of symptomatic dermatologic disease in homeless patients at a shelter-based clinic. Our Dermatol Online.

[11] Shahriari N, Torre K, Payette M (2017). Dermatologic conditions in a shelter-based homeless population: lssons learned from a medical student-run dermatology clinic. Conn Med.

[12] Hester T, Thomas R, Cederna J, Peterson AM, Brown J, Johnson TM, Cha KB (2021). Increasing access to specialized dermatology care: a retrospective study investigating clinical operation and impact of a university-affiliated free clinic. Dermatol Ther (Heidelb).

[13] Truong A, Secrest AM, Zhang M (2021). A survey of dermatologic health-related quality of life and resource access in patients experiencing homelessness. J Am Acad Dermatol.

[14] Sacheli R, Harag S, Dehavay F (2020). Belgian National Survey on tinea capitis: epidemiological considerations and highlight of terbinafine-resistant T. mentagrophytes with a mutation on SQLE gene. J Fungi (Basel).

[15] Tey HL, Tan AS, Chan YC (2011). Meta-analysis of randomized, controlled trials comparing griseofulvin and terbinafine in the treatment of tinea capitis. J Am Acad Dermatol.

[16] Souissi A, Ben Lagha I, Toukabri N, Mama M, Mokni M (2018). Morse code-like hairs in tinea capitis disappear after successful treatment. Int J Dermatol.

[17] Abdel-Rahman SM (2017). Genetic predictors of susceptibility to dermatophytoses. Mycopathologia.

[18] Dessinioti C, Katsambas A (2013). Seborrheic dermatitis: etiology, risk factors, and treatments: facts and controversies. Clin Dermatol.

[19] Honnavar P, Chakrabarti A, Prasad GS, Singh P, Dogra S, Rudramurthy SM (2017). β-Endorphin enhances the phospholipase activity of the dandruff causing fungi Malassezia globosa and Malassezia restricta. Med Mycol.

[20] Bonifaz A, Ramírez-Tamayo T, Saúl A (2003). Tinea barbae (tinea sycosis): experience with nine cases. J Dermatol.

[21] Adams BB (2002). Tinea corporis gladiatorum. J Am Acad Dermatol.

[22] Ely JW, Rosenfeld S, Seabury Stone M (2014). Diagnosis and management of tinea infections. Am Fam Physician.

[23] Gupta AK, Chaudhry M, Elewski B (2003). Tinea corporis, tinea cruris, tinea nigra, and piedra. Dermatol Clin.

[24] Singh S, Verma P, Chandra U, Tiwary NK (2019). Risk factors for chronic and chronic-relapsing tinea corporis, tinea cruris and tinea faciei: Results of a case-control study. Indian J Dermatol Venereol Leprol.

[25] Choi FD, Juhasz ML, Atanaskova Mesinkovska N (2019). Topical ketoconazole: a systematic review of current dermatological applications and future developments. J Dermatolog Treat.

[26] Brandi N, Starace M, Alessandrini A, Piraccini BM (2019). Tinea versicolor of the neck as side effect of topical steroids for alopecia areata. J Dermatolog Treat.

[27] Singh S, Sondhi P, Yadav S, Ali F (2017). Tinea barbae presenting as kerion. Indian J Dermatol Venereol Leprol.

[28] Yin X, Du X, Zhang H (2011). A case of tinea barbae due to Trichophyton rubrum infection by autoinoculation from the infected fingernails. Mycoses.

[29] Rajagopalan M, Inamadar A, Mittal A (2018). Expert Consensus on The Management of Dermatophytosis in India (ECTODERM India). BMC Dermatol.

[30] Leibler JH, Liebschutz JM, Keosaian J, Stewart C, Monteiro J, Woodruff A, Stein MD (2019). Homelessness, personal hygiene, and MRSA nasal colonization among persons who inject drugs. J Urban Health.

[31] Jones CL (1990). Foot care for the homeless. J Am Podiatr Med Assoc.

[32] Stratigos AJ, Katsambas AD (2003). Medical and cutaneous disorders associated with homelessness. Skinmed.

[33] (2012). LiverTox: Clinical and Research Information on Drug-Induced Liver Injury [Internet]. https://www.ncbi.nlm.nih.gov/books/NBK548617/.

[34] Taheri A, Davis SA, Huang KE, Feldman SR (2015). Onychomycosis treatment in the United States. Cutis.

[35] Ilkit M, Durdu M (2015). Tinea pedis: the etiology and global epidemiology of a common fungal infection. Crit Rev Microbiol.

[36] Drago L, Micali G, Papini M, Piraccini BM, Veraldi S (2017). Management of mycoses in daily practice. G Ital Dermatol Venereol.

[37] Chetana K, Menon R, David BG (2018). Onychoscopic evaluation of onychomycosis in a tertiary care teaching hospital: a cross-sectional study from South India. Int J Dermatol.

[38] Wollina U, Nenoff P, Haroske G, Haenssle HA (2016). The diagnosis and treatment of nail disorders. Dtsch Arztebl Int.

[39] Salakshna N, Bunyaratavej S, Matthapan L, Lertrujiwanit K, Leeyaphan C (2018). A cohort study of risk factors, clinical presentations, and outcomes for dermatophyte, nondermatophyte, and mixed toenail infections. J Am Acad Dermatol.

[40] Hanna S, Andriessen A, Beecker J (2018). Clinical insights about onychomycosis and its treatment: a consensus. J Drugs Dermatol.

[41] Petrovich JC, Hunt JJ, North CS, Pollio DE, Roark Murphy E (2020). Comparing unsheltered and sheltered homeless: demographics, health services use and predictors of health services use. Community Ment Health J.

[42] Rodriguez DA (2015). Efinaconazole topical solution, 10%, for the treatment of mild and moderate toenail onychomycosis. J Clin Aesthet Dermatol.

[43] Szepietowski JC, Reich A, Garlowska E, Kulig M, Baran E (2006). Factors influencing coexistence of toenail onychomycosis with tinea pedis and other dermatomycoses: a survey of 2761 patients. Arch Dermatol.

[44] Sahuquillo Torralba A, Navarro Mira MÁ, Botella Estrada R (2017). Inflammatory tinea manuum: the importance of pustules. Med Clin (Barc).

[45] Browning JC (2009). An update on pityriasis rosea and other similar childhood exanthems. Curr Opin Pediatr.

[46] Rotta I, Sanchez A, Gonçalves PR, Otuki MF, Correr CJ (2012). Efficacy and safety of topical antifungals in the treatment of dermatomycosis: a systematic review. Br J Dermatol.

[47] van Zuuren EJ, Fedorowicz Z, El-Gohary M (2015). Evidence-based topical treatments for tinea cruris and tinea corporis: a summary of a Cochrane systematic review. Br J Dermatol.

[48] Rosen T (2016). Mycological considerations in the topical treatment of superficial fungal infections. J Drugs Dermatol.

[49] Zhan P, Ge YP, Lu XL, She XD, Li ZH, Liu WD (2010). A case-control analysis and laboratory study of the two feet-one hand syndrome in two dermatology hospitals in China. Clin Exp Dermatol.

[50] Augustin M, Kirsten N, Körber A (2019). Prevalence, predictors and comorbidity of dry skin in the general population. J Eur Acad Dermatol Venereol.

[51] Kabtani J, Diongue K, Dione JN (2021). Real-time PCR assay for the detection of dermatophytes: Comparison between an in-house method and a commercial kit for the diagnosis of dermatophytoses in patients from Dakar, Senegal. J Fungi (Basel).

[52] Gaitanis G, Magiatis P, Hantschke M, Bassukas ID, Velegraki A (2012). The Malassezia genus in skin and systemic diseases. Clin Microbiol Rev.

[53] (1988). Homelessness, health, and human needs. Institute of Medicine (US) Committee on Health Care for Homeless People.

[54] Drake LA, Dinehart SM, Farmer ER (1996). Guidelines of care for superficial mycotic infections of the skin: tinea corporis, tinea cruris, tinea faciei, tinea manuum, and tinea pedis. Guidelines/Outcomes Committee. American Academy of Dermatology. J Am Acad Dermatol.

[55] Tirado-Sánchez A, Bonifaz A (2018). Tinea capitis: current review of the literature. Curr Fungal Infect Rep.

[56] Gómez-Moyano E, Martínez Pilar L, Vera Casaño A (2021). Correlation between dermoscopy and direct microscopy in Tineabarbae. J Mycol Med.

